# Drug-Drug Interaction Predicting by Neural Network Using Integrated Similarity

**DOI:** 10.1038/s41598-019-50121-3

**Published:** 2019-09-20

**Authors:** Narjes Rohani, Changiz Eslahchi

**Affiliations:** 1 Department of Computer Sciences, Faculty of Mathematics, Shahid Beheshti University, G.C, Tehran, 1983969411 Iran; 2 School of Biological Sciences, Institute for Research in Fundamental Sciences (IPM), Tehran, 193955746 Iran

**Keywords:** Data mining, Machine learning

## Abstract

Drug-Drug Interaction (DDI) prediction is one of the most critical issues in drug development and health. Proposing appropriate computational methods for predicting unknown DDI with high precision is challenging. We proposed "NDD: Neural network-based method for drug-drug interaction prediction" for predicting unknown DDIs using various information about drugs. Multiple drug similarities based on drug substructure, target, side effect, off-label side effect, pathway, transporter, and indication data are calculated. At first, NDD uses a heuristic similarity selection process and then integrates the selected similarities with a nonlinear similarity fusion method to achieve high-level features. Afterward, it uses a neural network for interaction prediction. The similarity selection and similarity integration parts of NDD have been proposed in previous studies of other problems. Our novelty is to combine these parts with new neural network architecture and apply these approaches in the context of DDI prediction. We compared NDD with six machine learning classifiers and six state-of-the-art graph-based methods on three benchmark datasets. NDD achieved superior performance in cross-validation with AUPR ranging from 0.830 to 0.947, AUC from 0.954 to 0.994 and F-measure from 0.772 to 0.902. Moreover, cumulative evidence in case studies on numerous drug pairs, further confirm the ability of NDD to predict unknown DDIs. The evaluations corroborate that NDD is an efficient method for predicting unknown DDIs. The data and implementation of NDD are available at https://github.com/nrohani/NDD.

## Introduction

DDIs are known as the unwanted side effects resulting from the concurrent consumption of two or more drugs^1–3^. When a doctor prescribes several drugs simultaneously for a patient, DDIs may cause irreparable side effects. The effects of drugs on each other may lead to other illnesses or even death. These side effects are particularly noticeable in adult people and cancer patients who take lots of drugs daily^4,5^.

Due to the importance of predicting DDIs in human health, industry and the economy, and the substantial amount of cost and time of traditional experimental approaches^6^, accurate computational methods for predicting DDI are in need. There is a huge number of biomedical information besides the development of computational approaches. For example, DrugBank is one of the most credible databases of known DDI^7–9^, which contains more than 300,000 DDIs. Nevertheless, this amount of interaction data is less than 1% of the total drug pairs exist in DrugBank. In the last decade, many computational methods have been developed to address this issue and overcome these limitations^10–13^. Vilar *et al*. developed a model to predict DDIs based on Interaction Profile Fingerprint (IPF)^11^. Quite simply, the interaction probability matrix was computed by multiplying the DDI matrix by the IPF matrix. Afterward, Lu *et al*. proposed a computational framework by applying matrix perturbation, based on the hypothesis that by removing the edges randomly from DDI network, the eigenvectors of the adjacent matrix of the network does not change^14^. Unfortunately, these two methods employ no other data about drugs, except known DDIs. Assuming that similar drugs may have almost similar interactions, a new trend of similarity-based methods was followed in studies. Vilar *et al*.^12^ presented a neighbor recommender method by utilizing substructure similarity of drugs. Relying on the Vilar’s framework, Zhang *et al*. constructed a weighted similarity network that is labeled based on interaction with each of drugs^13^ and applied an integrative label propagation method via the random walk on the network to estimate the potential DDIs. However, this predicting framework only considered three types of similarities for predicting DDI via label propagation, namely substructure-based, side effect-based, and offside effect-based label propagation models^13^. Concerning the hypothesis that each type of drug data may assist in disclosing the patterns of interactions, a new inclination toward ensemble methods had emerged. In this manner, Gottlieb *et al*.^15^ made use of seven drug-drug similarity measures and combined every two similarities and calculated 49 features for each drug pair and predict novel DDIs by Logistic Regression (LR). Afterward, a Heterogeneous Network-Assisted Inference (HNAI) framework was proposed^16^ by Chen *et al*., which applied five predictive models (Naive Bayes (NB), Decision Tree (DT), K-Nearest Neighbor (KNN), logistic regression, and Support Vector Machine(SVM)). In HNAI, drug pair similarities were calculated using four features: phenotypic, therapeutic, chemical, and genomic similarity. Zhang *et al*.^17^ have proposed an ensemble method that uses eight types of drug similarities and calculates six other DDI network-based drug similarities. It applies both the neighbor recommender method and the label propagation method on each of these 14 similarity type, that yields 28 models, as well as a matrix perturbation method on DDI network. At last, two ensemble methods with the weighted average ensemble rule and the classifier ensemble rule for integrating methods are adopted to aggregates these 29 models for predicting DDIs.

Liu *et al*.^18^ have proposed a Dependency-based Convolutional Neural Network (DCNN) for drug-drug interaction extraction. DCNN is a text-mining approach which predicts DDIs based on unstructured biomedical literature and the existing knowledge bases. It applies convolution layers on word sequences as well as dependency parsing trees of candidate DDIs for adjacent words. Ryu *et al*.^19^ proposed DeepDDI, which is a combination of the structural similarity profile generation pipeline and Deep Neural Network (DNN). DeepDDI predicts DDIs from chemical structures and names of drugs in pairs. It has various implications for adverse drug events such as prediction of potential causal mechanism and using them for output sentences. Lim *et al*.^20^ designed a DDI extraction model using a recursive neural network based approach which is a variation of the binary tree-LSTM model. This natural language processing model uses syntactical features of the parse tree to extract information from the DDI-related sentences. Wang *et al*.^21^ designed a DNN model to predicts the potential adverse drug reactions that take three types of features as the input: the chemical properties, biological properties, and information from the literature. This model considers drug relationships with the word-embedding method for analyzing substantial biomedical literature.

A new approach in this field is network pharmacology which identifies the synergistic interaction of drugs based on a specific disease. Li *et al*.^22^ have proposed NIMS method which constructs a disease-specific biological network between therapeutic targets. The background assumption of NIMS is that the interaction of drugs can be transferred as the interaction between targets or responsive gene products of drugs. NIMS utilizes this disease-specific network to calculate the synergistic relationship between each pair of drugs in the treatment of the disease. Guo *et al*.^23^ have shown that detecting synergistic combinations of compounds is efficient in the treatment of Inflammation-Induced Tumorigenesis (IIT). Since ITT is caused by major mutations in genes involved in proliferation, immune response, and metabolism^24^, synergistic drug combinations are useful in its treatment. Guo *et al*.^23^ used synergistic modules in differential gene interaction network to predict a set of four drugs that can effectively inhibit IIT.

Although previous methods had great advances, more prediction accuracy is still needed. We developed a method to overcome this issue via similarity selection, similarity fusion, and neural network. For similarity selection, a heuristic method was proposed by Olayan *et al*.^25^ in the context of drug-target interaction prediction. This method empowers making use of a high informative subset of data. Integrating more heterogeneous data is also helpful in enhancing the prediction performance. Similarity Network Fusion (SNF)^26^ is a competent method to integrate various similarities which is used in numerous biological contexts^25,27,28^. The neural network is a strongly developed approach that provides satisfactory solutions, especially for large datasets and nonlinear analyzes^29^ which is widely used in critical problems^30–32^.

In this study, we developed NDD that utilizes the neural network model along with similarity selection and fusion methods to take advantage of nonlinear analysis and professional feature extraction to improve the DDI prediction accuracy. NDD is a multi-step pipeline. In the first step, it obtains information of various drug similarities (chemical, target-based, Gene Ontology (GO), side effect, off-label side effect, pathway, Anatomical Therapeutic Chemical (ATC), ligand, transporter, indication, and Gaussian Interaction Profile (GIP) similarities) and their interaction data from different datasets. Then it selects the most informative and less redundant subset of similarity types by a heuristic process that proposed by Olayan *et al*.^25^. In the next step, the selected similarity types are integrated by a non-linear similarity fusion method called SNF^26^. Finally, the integrated similarity matrix, in addition to the interaction data is used for training the neural network. NDD can provide an accurate framework for predicting new DDIs. NDD utilizes previous methods in similarity selection and similarity fusion, but the architecture of the neural network is novel and highly tuned for this problem. Moreover, the previously proposed parts of NDD has not been used in DDI prediction context. Another novelty of this work is the combination of these steps with the neural network. All parts of NDD are described elaborately in the Materials and Methods section. We compared our method with two categories of methods, including graph-based methods and machine learning models via stratified five-fold cross-validation. To further demonstrate NDD’s ability in predicting unknown DDIs, case studies on several drugs were investigated. These results confirm that NDD is an efficient method for accurate DDI prediction.

## Results

For validating the robustness of NDD, we investigated it on three datasets (DS1, DS2, and DS3). The detailed description of these datasets is provided in the Materials and Methods section. We compared our method with common machine learning classifiers such as random forest^33^, logistic regression^34^, adaptive boosting^35^, linear discriminant analysis (LDA)^36^, quadratic discriminant analysis (QDA)^37^, and k-nearest neighbor^38^ on these datasets. Meanwhile, we also evaluated the performance of six state-of-the-art graph-based methods including Vilar’s substructure-based model^11^, substructure-based label propagation model^13^, side effect-based label propagation model^13^, offside effect-based label propagation model^13^, weighted average ensemble method^17^, classifier ensemble method^17^ on DS1, DS2, and DS3.

The mentioned machine learning methods use just one similarity in their processes. To have a fair comparison between NDD and these methods, we applied similarity selection and fusion described in Subsections 4.4 and 4.5 on all similarity types and used the integrated similarity matrix as the inputs for machine learning methods.

### Evaluation criteria

In this study, we classify drug pairs to be interacting or not. So, we exploit commonly used metrics in classification, including precision, recall, F-measure, AUC, and AUPR, defined as follows.

1 $$precision=\frac{TP}{TP+FP}$$

2 $$recall=\frac{TP}{TP+FN}$$

3 $$F-measure=\frac{2\cdot precision\cdot recall}{precision+recall}$$

Where TP, TN, FP, and FN stand for True Positive, True Negative, False Positive, and False Negative. Precision is the fraction of correct predicted interactions among all predicted interactions, while recall is the fraction of correct predicted interactions among all true interactions. Precision and recall have a trade-off; thus, improving one of them may lead to a reduction in another. Therefore, utilizing F-measure, which is the geometric mean of precision and recall, is more reasonable. The reported values for precision, recall, and F-measure of each method is based on the best value of the threshold for the output.

It should be noted that if the interaction of two drugs is assigned to zero, it denotes that no evidence of their interaction has been found yet; thus, they may interact with each other. So we cannot identify TN and FP pairs correctly. The training process requires both positive and negative samples. Therefore, some of the zero assigned pairs are considered as non-interactive pairs in the training model. So every method may have some FP in its evaluations. This leads to a reduction in calculated precision and F-measure, while the real values of precision and F-measure may be higher.

Since the values of precision, recall, and F-measure is dependent to the value of the threshold, we also evaluate methods via AUC which is the area under the receiver operating characteristic (ROC) curve, and AUPR, that is the area under the precision-recall curve. These criteria indicate the efficiency of methods independent of the threshold value. In cases that the fraction of negative samples and positive samples are not equal, AUPR is the fairer criterion for evaluation.

The assessments of method performances are conducted by stratified five-fold cross-validation. The procedure of stratified five-fold cross-validation is repeated 20 times and averaged to ensure low-variance and unbiased evaluations.

### Comparison of NDD with other methods on DS1

We first evaluated NDD on DS1 in comparison to other methods. Diverse types of machine learning classifiers such as linear, nonlinear, tree-based, kernel-based, and ensemble approaches are considered in our evaluations.

A summary of computed criteria for all methods is presented in Table 1. It is not very surprising that the classifier ensemble method has the highest performance in terms of most criteria since it is designed and proposed for this particular dataset and this is the dataset that is compiled by the one who proposed the classifier ensemble method. Nevertheless, NDD with AUC of 0.954 (p-value = 9.105e-15, t-test), AUPR of 0.922 (p-value = 1.678e-23, t-test), F-measure of 0.835 (p-value = 1.127e-23, t-test), recall of 0.828 (p-value = 7.504e-21, t-test), and precision of 0.831 (p-value = 4.203e-21, t-test), had infinitesimal differences in all criteria compared to the classifier ensemble method. Furthermore, NDD achieved almost similar performance in comparison with the weighted average ensemble method, which is also designed specifically for DS1. Besides, NDD performs better than individual predictors (Vilar’s substructure-based model, substructure-based label propagation model, side effect-based label propagation model, and offside effect-based label propagation). Moreover, NDD outperforms machine learning classifiers (RF, LR, adaptive boosting, LDA, QDA, and KNN) in terms of all criteria. The results indicate that making use of the neural network, gives good discriminatory features and general machine learning models might not well handle to find hidden interactions.

**Table 1:** Performance comparison of all methods on DS1.

Method	AUC	AUPR	F-measure	Recall	Precision
Substructure-based label propagation model	0.937	0.901	0.804	0.797	0.811
Side-effect-based label propagation model	0.936	0.903	0.806	0.793	0.820
Offside-effect-based label propagation model	0.937	0.904	0.809	0.795	0.823
Vilar’s substructure-based model	0.936	0.902	0.804	0.797	0.812
Classifier ensemble method	**0.956**	**0.928**	**0.836**	0.827	**0.843**
Weighted average ensemble method	0.948	0.919	0.831	0.835	0.826
NDD	0.954	0.922	0.835	0.836	0.833
RF	0.830	0.693	0.666	0.738	0.607
LR	0.941	0.905	0.812	0.810	0.818
Adaptive boosting	0.722	0.587	0.558	0.572	0.546
LDA	0.935	0.898	0.801	0.800	0.803
QDA	0.857	0.802	0.751	**0.912**	0.638
KNN	0.730	0.134	0.080	0.062	0.098

The best value of each criterion is shown in bold.

Although Vilar *et al*. considered only substructure similarity for predicting DDI via neighbor recommender^11^ and Zhang *et al*. considers three types of similarities for predicting DDI via label propagation^13^, we further applied neighbor recommender and label propagation on other types of similarity as well as the integrated similarity matrix. The results are presented in supplementary material (see Supplementary File 1). One can conclude the most important similarity type for each of these methods by comparing the results.

### Comparison of NDD performance on different similarity types in DS1

To investigate the impact of different similarity types on NDD performance, we ignored the selection section 4.4 and fusion section 4.5 of NDD and just applied its neural network on the features provided with each similarity matrix.

From Table 2, we can see that NDD has the poorest performance when it is provided with only the chemical similarity. These results are in accordance with the selected subset of similarities after applying the procedure introduced in Subsection 4.4. The subset of similarities that is selected includes all similarity types except chemical similarity since its entropy is greater than the threshold 0.6; so it is excluded from the selected list.

**Table 2:** Comparison of NDD performance on different similarity types.

Method	Similarity	AUC	AUPR	F-measure	Recall	Precision
NDD	chemical	0.631	0.455	0.527	0.899	0.373
NDD	target	0.787	0.642	0.617	0.721	0.540
NDD	transporter	0.682	0.568	0.519	0.945	0.358
NDD	enzyme	0.734	0.599	0.552	0.579	0.529
NDD	pathway	0.767	0.623	0.587	0.650	0.536
NDD	indication	**0.802**	**0.654**	**0.632**	0.740	**0.551**
NDD	side effect	0.778	0.601	0.619	0.748	0.528
NDD	offside effect	0.782	0.606	0.617	**0.764**	0.517

The best value of each criterion is shown in bold.

We further compare each line of Table 2 with the seventh line of Table 1, to evaluate the fusion process. The superiority of NDD performance on integrated similarity over its performance when provided with each of similarities, completely verify that making use of integrated similarity matrices leads to discover more discriminant features and significantly improves the prediction performance.

In addition, we repeat this evaluation for each similarity types in DS3. The results reconfirm that the integration of similarities yields more discriminant features in predicting DDIs.

### Comparison of NDD with other methods on DS2

To test the reliability and robustness of NDD, we also assessed its performance on another dataset that is compiled by Wan *et al*.^39^. This dataset contains only the chemical similarity of drugs.

As shown in Table 3, the highest performance in terms of all criteria is achieved by NDD. NDD outperforms both the graph-based methods (the first six rows) and machine learning methods (the last six rows). Almost all methods failed to yield promising results. The difference between NDD performance with other methods in terms of AUPR, F-measure, recall, and precision is outstanding. Even the performance of ensemble methods (the classifier ensemble method and the weighted average ensemble method), that make use of combining the results of diverse approaches, are not comparable to NDD. The striking performance of NDD on DS2, indicates the great extent of complexity of relations in this dataset; thus, NDD is very promising in extracting very discriminant features that can easily discover hidden DDIs in situations that other state-of-the-art methods do not perform well.

**Table 3:** Performance comparison of all methods on DS2.

Method	AUC	AUPR	F-measure	Recall	Precision
Substructure-based label propagation model	0.788	0.208	0.294	0.537	0.197
Vilar’s substructure-based model	0.810	0.244	0.312	0.479	0.232
Classifier ensemble method	0.936	0.487	0.553	0.689	0.462
Weighted average ensemble method	0.646	0.440	0.15	0.226	0.118
NDD	**0.994**	**0.890**	**0.825**	**0.804**	**0.847**
RF	0.982	0.812	0.747	0.713	0.785
LR	0.911	0.251	0.318	0.397	0.268
Adaptive boosting	0.904	0.185	0.266	0.359	0.211
LDA	0.894	0.215	0.295	0.407	0.231
QDA	0.926	0.466	0.174	0.875	0.096
KNN	0.927	0.785	0.602	0.445	0.932

The best value of each criterion is shown in bold.

### Comparison of NDD with other methods on CYP interactions of DS3

For further testing the efficiency of NDD, we evaluated our proposed method on DS3. Since this dataset contains two types of interactions, the comparisons were conducted in two steps.

The computed criteria for results of algorithms in predicting CYP (the Cytochrome P450 involved DDIs) interaction are illustrated in Table 4. It can be concluded that NDD outperforms other graph-based methods. NDD succeeded to improve AUC by 5%, and AUPR by 29%, which implicates the better structure of NDD in predicting CYP DDIs. It is noteworthy that, due to the lower proportion of positive samples in CYP interactions in comparison with NCYP (the DDIs without involving Cytochrome P450) interactions, the performance of methods declines in these types of interactions.

**Table 4:** Performance comparison of all methods on CYP interactions of DS3.

Method	AUC	AUPR	F-measure	Recall	Precision
Substructure-based label propagation model	0.952	0.126	0.206	0.278	0.161
Side-effect-based label propagation model	0.953	0.120	0.199	0.278	0.156
Vilar’s substructure-based model	0.953	0.126	0.196	0.279	0.152
Classifier ensemble method	0.990	0.541	0.553	0.566	0.546
Weighted average ensemble method	0.695	0.484	0.198	0.201	0.201
NDD	**0.994**	**0.830**	**0.772**	**0.770**	**0.775**
RF	0.737	0.092	0.161	0.216	0.132
LR	0.977	0.487	0.524	0.589	0.475
Adaptive boosting	0.830	0.143	0.215	0.259	0.185
LDA	0.953	0.327	0.388	0.363	0.425
QDA	0.709	0.317	0.259	0.446	0.184
KNN	0.590	0.064	0.039	0.008	0.190

The best value of each criterion is shown in bold.

### Comparison of NDD with other methods on NCYP interactions of DS3

In this section, we investigate the method performance on NCYP interactions. Through these evaluations, which are summarized in Table 5, the well-structured framework of NDD is verified. There is a significant improvement in AUPR, F-measure, precision, and recall by NDD method. Moreover, NDD obtained the best AUC in NCYP DDI prediction. For instance, AUPR has improved by 19%. Based on these results, we found the significant supremacy of NDD in terms of AUC as well as AUPR and F-measure, which are so consistent with the evaluations of previous sections and further confirm the capability of NDD in predicting NCYP DDIs.

**Table 5:** Performance comparison of all methods on NCYP interactions of DS3.

Method	AUC	AUPR	F-measure	Recall	Precision
Substructure-based label propagation model	0.890	0.159	0.216	0.379	0.153
Side-effect-based label propagation model	0.895	0.181	0.234	0.285	0.208
Vilar’s substructure-based model	0.904	0.295	0.248	0.383	0.183
Classifier ensemble method	0.986	0.756	0.708	0.702	0.714
Weighted average ensemble method	0.974	0.599	0.587	0.584	0.591
NDD	**0.992**	**0.947**	**0.902**	**0.884**	**0.918**
RF	0.889	0.167	0.242	0.411	0.168
LR	0.916	0.472	0.506	0.571	0.454
Adaptive boosting	0.709	0.150	0.193	0.358	0.141
LDA	0.889	0.414	0.456	0.501	0.419
QDA	0.536	0.260	0.132	0.080	0.387
KNN	0.603	0.235	0.134	0.229	0.094

The best value of each criterion is shown in bold.

### Case studies

To further demonstrate NDD’s ability for predicting unknown DDIs, we investigated FPs on the DS1, in reliable databases and literature. Inspecting the FP drug pairs that obtain high probabilities of having interaction, authenticated that there is a substantial body of evidence in the valid databases and published papers for almost all of such FPs. The top ten predicted DDIs are presented in Table 6, from which six interactions now exist in the DrugBank database, but they were labeled zero in our training samples. There is a great number of confirmations for DDIs that are newly predicted by NDD; we list numerous top predicted DDIs along with their evidence in the Supplementary File 2. Furthermore, a brief description of evidences for two case studies that their predicted interactions probability were above %90 is presented in Supplementary File 3.

**Table 6:** Top ten predicted interactions (confirmed interactions by DrugBank database is shown in bold).

Rank	ID1	ID2	Drug Name 1	Drug Name 2
1	**DB00642**	**DB01331**	Pemetrexed	Cefoxitin
2	**DB00642**	**DB01060**	Pemetrexed	Amoxicillin
3	**DB00633**	**DB01183**	Dexmedetomidine	Naloxone
4	DB00633	DB00361	Dexmedetomidine	Vinorelbine
5	DB00535	DB00373	Cefdinir	Timolol
6	**DB01236**	**DB01586**	Sevoflurane	Ursodeoxycholic acid
7	DB01236	DB00415	Sevoflurane	Ampicillin
8	**DB00742**	**DB00441**	Mannitol	Gemcitabine
9	**DB00585**	**DB01577**	Nizatidine	Methamphetamine
10	DB01136	DB00952	Carvedilol	Naratriptan

## Discussion

For predicting novel DDIs, a computational method is proposed based on similarity selection and fusion in addition to using the neural network. Its performance is very surprising, especially on datasets that had more complexity and required high-order features for eliciting hidden interactions. Its performance was significant in comparison with the graph-based and machine learning methods. Several reasons account for the high performance of NDD:

1. It takes advantage of different types of similarity matrices; thus, it gets diverse information from different aspects of drug pairs that yield an inclusive insight about them.
2. It utilizes a similarity selection approach and a fusion procedure as refinement and abstraction of whole data to avoid noise, reduce redundant information, and exclude random-like data.
3. Two drugs may be considered similar due to the lack of information. This issue may occur for each type of similarities. NDD uses similarity integration method that can help to overcome this limitation.
4. Neural network can automatically process the input features and elicit high-level features from them that leads to better prediction performance.

The performance of NDD was evaluated in two mechanisms: 1- comparing results of NDD with other methods and conducting t-test to test the statistical significance of NDD performance in terms of evaluation criteria scores. 2- case studies on FP predictions. There were many potential DDIs predicted by our method that has a body of evidence in the literature and credible databases. However, many other FPs are expected to be verified by reliable resources in the near future.

It should be noted that NDD needs much time to train. This is due to the fact that the number of trainable parameters of the neural network is high. Furthermore, it is difficult to list out all possible neural network architecture, and it causes the difficulty to find the optimal architecture. Running neural network-based methods needs powerful hardware. In addition to these limitations, for constructing training data, we require both interacting drug pairs and non-interacting drug pairs to train the models both with positive and negative samples and to avoid over-fitting. Nevertheless, it is almost impossible to verify that a negative drug pair is a non-interacting pair or interacting pair that is not discovered yet.

A strategy to decrease method complexity is dimension reduction; i.e. each column of the integrated similarity matrix can be considered as a feature. We applied common feature selection method Chi2^40^ and multiple state-of-the-art feature extraction methods such as Non-negative Matrix Factorization (NMF), Principle Component Analysis (PCA), and Stacked Auto-Encoder (SAE) with the various number of features. Moreover, we tune the hyperparameters of the neural network based on the selected features to obtain optimal results. The calculated evaluation criteria are presented in Supplementary File 4. By comparing these results with typical NDD performance in Table 1, it can be concluded that none of the feature extraction methods, nor the feature selection method can improve the performance of the method. There is an interesting issue that the values of AUPR and AUC decreases as the number of (selected/extracted) features reduces. Note that these methods were applied to the columns of the integrated matrix and the integrated matrix was obtained by integrating a subset of similarity matrices with high information and less redundancy. Putting all these together, it can be inferred that reducing its dimensional leads to missing informative data and this lack of information causes poor performance.

## Materials and Methods

### Datasets

To verify the robustness of NDD, we validate it on different benchmarks with DDI and similarity data. We used the benchmark datasets that used in previous studies^15,17,39^.

The first benchmark (DS1) contains 548 drugs, 97168 interactions, and eight types of similarities based on substructure data, target data, enzyme data, transporter data, pathway data, indication data, side effect data, and offside effect data^17^. The second benchmark (DS2) contains 707 drugs, 34412 interactions, and a chemical similarity matrix^39^. The third dataset (DS3) consists of 807 drugs and seven types of similarity matrices, four of which are based on ATC, chemical similarity, ligand-based chemical similarity, and side effects. Three more similarity measures are constructed based on drug target similarities such as sequence similarity, the distance on the Protein-Protein Interaction (PPI) network and GO annotations^15^. It should be noted that the third dataset contains two types of interaction: CYP and NCYP.

These data are collected from the following databases:

- *DrugBank*^7,41^: A reliable database that contains information about drugs such as drug targets, drug enzymes, drug interactions, and drug transporters.
- *SIDER*^42^: A side effect database containing information of adverse drug reactions, side effect and the indication of drugs.
- *KEGG*^43^: A valid database that contains protein pathways information. This database is used to obtain drug pathways by mapping drug targets.
- *PubChem*^44^: The best reference database for drugs structures.
- *OFFSIDES*^45^: A collection of off-label side effect information about drugs.

Further details about similarities and datasets are available in Table 7 and Supplementary File 5. All similarity and interaction matrices are provided in https://github.com/nrohani/NDD.

**Table 7:** Details of Benchmarks.

Benchmark Name	Reference	Number of Drugs	Number of Pairs	Number of Interactions	Number of Non-interactions	Number of Similarities	Similarity Types
DS1	Zhang *et al*.^17^	548	300304	97168	203136	8	Chemical, Target, Transporter, Enzyme, Pathway, Indication, Side effects, Offside effect
DS2	Wan *et al*.^39^	707	499849	34412	465437	1	Chemical
DS3: CYP	Gottlieb *et al*.^15^	807	651249	10078	641171	7	GO, Target, Ligand, Chemical, PPI Distance, Side effect, ATC
DS3: NCYP	Gottlieb *et al*.^15^	807	651249	40904	610345	7	GO, Target, Ligand, Chemical, PPI Distance, Side effect, ATC

### NDD overview

The steps of NDD is as follows:

1. Calculating drug similarities and GIP for each drug pair.
2. Selecting a subset of similarities with the most information and less redundancy.
3. Integrating the selected similarities to obtain an integrated similarity matrix that represents all information in one matrix.
4. For each drug pair, the corresponding rows of the integrated matrix are concatenated and fed to a two-layer neural network to classify. A sigmoid function is used in the output layer of the neural network to obtain the probability of the interaction between input drug pairs.

**Figure 1:**
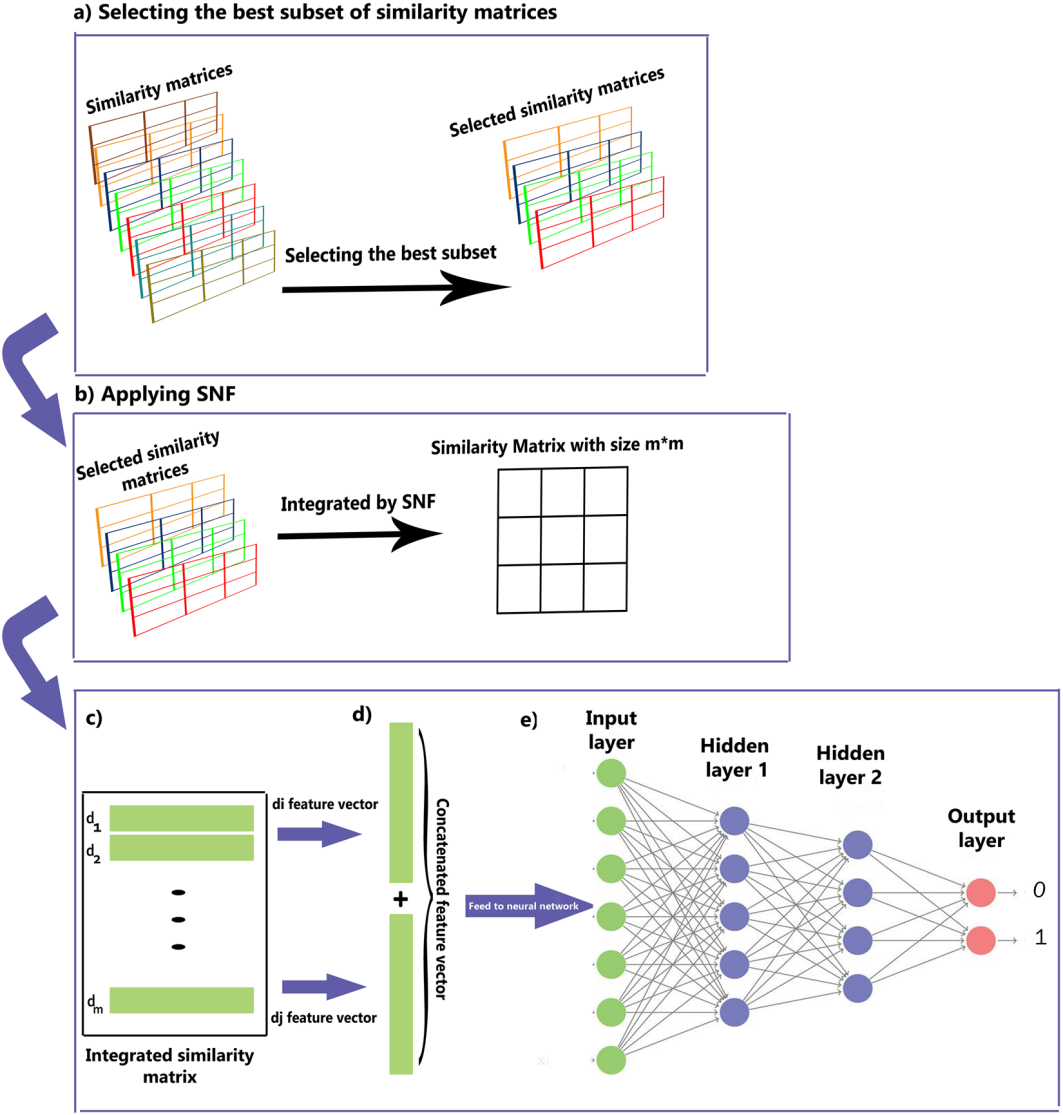
The scheme of NDD workflow. (**a**) Selecting the best subset of similarity matrices. (**b**) Applying SNF, a fusion method, to integrate all selected similarities into an m*m matrix where m is the number of drugs. (**c**) Every row in the integrated matrix, is the feature vector of its corresponding drug. (**d**) For each pair of the drugs, their feature vectors are concatenated in a vector and is considered as the input of a neural network. (**e**) The neural network is applied to the input vector to calculate the probability of interaction between input drug pair.

A scheme of the NDD is shown in Figure 1.

### Gaussian interaction profile

In addition to the mentioned drug similarities, we use GIP which is defined by Van *et al*.^46^. Let matrix *Y* = [*y*_*ij*_] be the interaction matrix that *y*_*ij*_∈{0, 1} that 1 indicates the existence of interaction between drugs *d*_*i*_ and *d*_*j*_ and 0 the otherwise. The GIP, for drugs *d*_*i*_ and *d*_*j*_ is defined as:

4 $$GI{P}_{d}({d}_{i},{d}_{j})=exp(-{\gamma }_{d}||{Y}_{i}-{Y}_{j}|{|}^{2})$$

where *Y*_*i*_ is the interaction profile of drug *i* with all drugs; in other words, *Y*_*i*_ is *i*th column of matrix *Y*. The parameter *γ*_*d*_ controls the bandwidth. We set

5 $${\gamma }_{d}={\tilde{\gamma }}_{d}/(\frac{1}{n}\mathop{\sum }\limits_{i=1}^{m}\,|{Y}_{{d}_{i}}{|}^{2})$$

In order to make GIP values, independent of the size of the dataset, we normalize them via dividing to the average number of interactions per drug. Here we set ${\tilde{\gamma }}_{d}=1c$ according to the previous research^25^. It should be noted that the GIP similarity is considered only for training data and not for the test data. Because it is assumed that the interaction information is not available for test data.

### Similarity selection

NDD uses multiple similarity matrices for drugs based on various similarity types as well as GIP. Before utilizing these similarities in the neural network model, it is essential to integrate different similarities. Due to the different amount of similarity information and their dependencies, combining all of these similarity matrices is not reasonable. It also may increase the noise in the final integrated similarity. Thus, we apply an efficient approach to select a subset of these matrices that is more informative and less redundant. Then, a similarity fusion method is used to integrate the chosen similarity matrices into one final integrated similarity.

We used the similarity selection heuristic process that is introduced by Olayan *et al*.^25^. It has several steps:

1. Calculating entropy of each matrix
2. Rank matrices based on their entropy
3. Calculate the pairwise distance of matrices
4. Final selection from the ranked list of matrices based on redundancy minimization

#### Calculating entropy

The entropy of each matrix demonstrates the extent of the carried information. Let *A* = [*a*_*ij*_]_*m*×*m*_ be the similarity matrix between *m* drugs. The entropy *E*_*i*_(*A*) for *i*th row is computed as follows:

6 $${E}_{i}(A)=-\,\sum _{j}\,{p}_{ij}\,\log \,{p}_{ij}$$

where

7 $${p}_{ij}=\frac{{a}_{ij}}{{\sum }_{k}\,{a}_{ik}}$$

The entropies of all rows are averaged to calculate the entropy of a matrix.

#### Rank matrices

The matrices are ranked in ascending order based on their entropy values. The entropy value indicates the amount of random information contained in the similarity matrix. Thus, matrices with high entropy have high random information and low values of entropy indicate less random information. So the similarity matrices with entropy values greater than *c*_1_ log(*m*) are removed. Constant *c*_1_ manages the level of entropy in the selected matrices. We examined several numbers for *c*_1_ in (0, 1) and evaluated the total performance of the model with these values. The best results were obtained with *c*_1_ = 0.6.

#### Calculate the pairwise distance

The similarity between two feature matrices *A* and *B* is defined as follows:

8 $$S(A,B)=\frac{1}{1+D(A,B)}$$

where *D*(*A*, *B*) is the Euclidean distance between *A* and *B* matrices, which is calculated as follows:

9 $$D(A,B)=\sqrt{\mathop{\sum }\limits_{i=1}^{m}\,\mathop{\sum }\limits_{j=1}^{m}\,{({a}_{ij}-{b}_{ij})}^{2}}$$

where *a*_*ij*_ and *b*_*ij*_ are the entries of *A* and *B* matrices, respectively.

#### Final selection

The selected subset of matrices is obtained by an iterative procedure. Suppose there exist *n* similarity matrices *A*_1_, *A*_2_, …, *A*_*n*_. In the first iteration, the set of selected matrices is empty and the set of ranked list is the one obtained by Subsection 4.4.2. In each iteration, the matrix with the least value of entropy from the ranked list *C* = *argmin*_*l*_ *E*(*A*_*l*_) is added to the selected set of matrices and all matrices *A*_*j*_ that has great similarity with *C*, *S*(*C*, *A*_*j*_) > *c*_2_, are eliminated from the ranked list. This procedure is iterated with the updated ranked list. It iterates until the ranked list is empty. Constant *c*_2_ is a threshold for the similarity of chosen matrices, which is subjectively set to 0.6 in our work. The value of this threshold was chosen by examining several numbers in (0, 1) and evaluating the performance of the model.

Eventually, this procedure selects a subset of similarity matrices that are highly informative (due to selecting matrices with low entropy) and have low redundancy (due to eliminating matrices with high similarity).

### Similarity network fusion

After selecting a reasonable subset of similarity matrices, we use the SNF method proposed by Wang *et al*.^26^ to integrate the selected matrices. It combines multiple similarity measures into a single fused similarity that carry an appropriate representation of all information. SNF applies an iterative nonlinear method that updates every similarity matrix according to the other matrices via KNN.

### Neural network model selection

To obtain an accurate neural network architecture, various networks were trained on datasets with different structures by nested cross-validation^47^. In nested cross-validation the hyperparameters are tuned via two cross-validations according to the following steps:

1. Setting the hyperparameters to some values.
2. Partitioning the data into three “folds” (sets).
3. Training the model using two folds with the hyperparameter values.
4. Testing the model on the remaining fold.
5. Performing steps 3 and 4 again and again; thus, every time one of the folds is considered as the test data.
6. Repeating steps 1 to 5 for all combinations of hyperparameter values.
7. Returning the combination of hyperparameter values that had the best performance.

In our model, we considered the following hyperparameter sets:

- Number of hidden layers: {1, 2, …, 5}
- Number of neurons in hidden layers: {100, 200, 300, 400, 500}
- Activation functions: {Rectified linear activation function(ReLU), hyperbolic tangent (tanh), and sigmoid}
- Dropout rate: {0.3, 0.5}

The best results were obtained with two hidden layers with 300 neurons in the first and 400 neurons in the second hidden layer with Dropout rate 0.5 for both layers. We used Dropout layer^48^ behind each layer to prevent the over-fitting problem. The output of each neuron in a layer is a nonlinear function *f* of all nodes in the previous layer. *f* is the ReLU, which is defined as the positive part of its argument:

10 $$f(x)={x}^{\ast }=\,{\rm{\max }}\,\{x,0\}$$

The final output is calculated using the sigmoid function, which is calculated as follows.

11 $$Sigmoid\,(x)=\frac{1}{1+{e}^{-x}}$$

The batch size was set to 200 and the epoch number was set to 20, 50, and 50 for DS1, DS2, and DS3, respectively, because the model yielded the best performance in these setting. At first, the weights were initialized with a normal distribution with a standard deviation of 0.05, and biases were initialized with a uniform distribution in (−1,0) which is common.

Afterward, the network is trained using interaction label information and input features to update weights and biases parameters. To train the model, we use the cross-entropy loss function (*C*.*E*)^49^ and Stochastic Gradient Descent (SGD)^50^ optimization with momentum value (0.9). NDD is implemented via Keras library^51^ in Python3.5. More details about the implementation of methods are presented in https://github.com/nrohani/NDD.

### Neural network for classification

After integrating selected similarities, we assigned label ‘1’ to all known DDI (positive samples) and 0 to others for every drug pairs. Then, data were divided into training data and test data with stratified five-fold cross-validation. Stratified five-fold cross-validation randomly divides data into five equal-sized sets such that the ratio of positive and negative pairs in all sets are equal. The training data were given to a neural network for predicting the label of each pair of drugs, which indicated whether it has a connection or not. Then, for each pair of drug *i* and drug *j*, the *i*th row of drug similarity matrix (i.e., the similarity data between drug i and all the other drugs) is concatenated to the jth row of drug similarity matrix (similar to the previous works^31,32,52^) and considered as a feature vector, which is fed to the neural network as an independent training sample. Then the network will be trained with the training set and the weights of the network will be updated. Once the training is accomplished, the trained neural network is implemented on test data to predict the interaction between drugs. The output is the probability of interaction between the input pairs. Two drugs that lead to a probability higher than the threshold are considered as potential interacting drugs.

## Supplementary information

Supplementary files

## Data Availability

Available at https://github.com/nrohani/NDD.
