# Supplementary material for: Drug-Drug Interaction Predicting by Neural Network Using Integrated Similarity
Source: Sci Rep. 2019 Sep 20;9:13645. doi: 10.1038/s41598-019-50121-3 (PMC6754439; doi:10.1038/s41598-019-50121-3)
Supplement: Supplementary file 1 — Supplementary files [file 41598_2019_50121_MOESM1_ESM.pdf]

Supplementary Information for

Drug-Drug Interaction Predicting by Neural Network Using Integrated Similarity

Narjes Rohani[1] and Changiz Eslahchi[2,1,*]

[1]Department of Computer Sciences, Faculty of Mathematics, Shahid Beheshti University, G.C. , Tehran, 1983963113, Iran
[2]School of Biological Sciences, Institute for research in fundamental sciences(IPM), Tehran, 193955746, Iran
[*]Ch-Eslahchi@sbu.ac.ir

Supplementary File #1 — The results of the neighbor recommender and label propagation on each types of similarity

Supplementary Tables S1 and S2 summarizes the calculated criteria for performance of neighbor recommender and label propagation trained on different similarity matrices in DS1.

Method	Similarity	AUC	AUPR	F-measure	Recall	Precision
NR	integrated	0.838	0.731	0.669	0.759	0.599
NR	chemical	0.936	0.902	0.804	0.797	0.812
NR	target	0.820	0.698	0.667	0.719	0.623
NR	transporter	0.734	0.637	0.596	0.647	0.550
NR	enzyme	0.756	0.670	0.599	0.690	0.531
NR	pathway	0.812	0.774	0.672	0.615	0.742
NR	indication	0.912	0.845	0.768	0.803	0.736
NR	side effects	0.936	0.901	0.803	0.795	0.813
NR	offside effect	0.940	0.913	0.837	0.839	0.843

Supplementary Table S 1. Performance of NR on different similarity matrices in DS1.

Method	Similarity	AUC	AUPR	F-measure	Recall	Precision
LP	integrated	0.898	0.831	0.742	0.780	0.708
LP	chemical	0.937	0.901	0.804	0.797	0.811
LP	target	0.851	0.794	0.703	0.683	0.627
LP	transporter	0.721	0.645	0.586	0.664	0.525
LP	enzyme	0.770	0.716	0.613	0.653	0.590
LP	pathway	0.810	0.779	0.675	0.606	0.767
LP	indication	0.940	0.911	0.818	0.808	0.828
LP	side effects	0.936	0.903	0.806	0.793	0.820
LP	offside effect	0.937	0.904	0.809	0.795	0.823

Supplementary Table S 2. Performance of LP on different similarity matrices in DS1.

Supplementary Tables S3, S4, S5 and S6 summarizes the calculated criteria for performance of neighbor recommender and label propagation trained on different similarity matrices in CYP and NCYP interactions of DS3.

Method	Similarity	AUC	AUPR	F-measure	Recall	Precision
NR	integrated	0.8871	0.100	0.1782	0.196	0.142
NR	ATC	0.93462	0.07316	0.13056	0.1573	0.1182
NR	Chemical	0.95274	0.12608	0.1964	0.27946	0.1525
NR	Distance on PPI	0.94812	0.10974	0.17442	0.18888	0.17174
NR	GO	0.95272	0.1235	0.1965	0.2719	0.1541
NR	Ligand	0.90548	0.0349	0.08326	0.13464	0.06096
NR	Target	0.94396	0.09098	0.15774	0.17638	0.1537
NR	Side effect	0.95102	0.09072	0.17144	0.23754	0.13578

Supplementary Table S 3. Performance of NR on different similarity matrices in CYP interactions of DS3.

Method	Similarity	AUC	AUPR	F-measure	Recall	Precision
LP	integrated	0.906	0.148	0.225	0.282	0.189
LP	ATC	0.94992	0.10684	0.17864	0.1984	0.16478
LP	Chemical	0.95256	0.12696	0.20644	0.28222	0.16358
LP	Distance on PPI	0.95216	0.1312	0.20672	0.2576	0.18308
LP	GO	0.95294	0.12608	0.2061	0.28698	0.1624
LP	Ligand	0.94712	0.09672	0.16478	0.2008	0.15184
LP	Target	0.95226	0.12692	0.1981	0.23614	0.1838
LP	Side effect	0.95324	0.12004	0.1992	0.27844	0.1569

Supplementary Table S 4. Performance of LP on different similarity matrices in CYP interactions of DS3.

Method	Similarity	AUC	AUPR	F-measure	Recall	Precision
NR	integrated	0.778	0.184	0.259	0.483	0.192
NR	ATC	0.94052	0.38662	0.4159	0.35186	0.51112
NR	Chemical	0.90392	0.19562	0.24828	0.38358	0.18366
NR	Distance on PPI	0.94436	0.3713	0.4149	0.43926	0.3936
NR	GO	0.9144	0.23408	0.28836	0.3494	0.24568
NR	Ligand	0.95058	0.40722	0.4375	0.44024	0.43602
NR	Target	0.95134	0.42558	0.445	0.43374	0.4574
NR	Side effect	0.92392	0.29786	0.34332	0.3448	0.34416

Supplementary Table S 5. Performance of NR on different similarity matrices in NCYP interactions of DS3.

Method	Similarity	AUC	AUPR	F-measure	Recall	Precision
LP	integrated	0.667	0.216	0.333	0.449	0.267
LP	ATC	0.92698	0.31984	0.37146	0.35212	0.39316
LP	Chemical	0.89046	0.1591	0.21636	0.37964	0.15314
LP	Distance on PPI	0.8993	0.19242	0.24148	0.2804	0.23194
LP	GO	0.88938	0.167	0.22092	0.33466	0.17868
LP	Ligand	0.93758	0.33404	0.37756	0.41718	0.34556
LP	Target	0.90266	0.2165	0.26842	0.30518	0.24436
LP	Side effect	0.89534	0.18118	0.23442	0.28542	0.2081

Supplementary Table S 6. Performance of LP on different similarity matrices in NCYP interactions of DS3.

Supplementary File #2 — Body of evidence for the case studies

Drug1 ID	Drug2 ID	Drug1 name	Drug2 name	Evidence
DB00642ă	DB01331ă	Pemetrexed	Cefoxitin	DrugBank
DB00642ă	DB01060ă	Pemetrexed	Amoxicillin	DrugBank
DB00633ă	DB01183ă	Dexmedetomidine	Naloxone	DrugBank
DB00633ă	DB00361ă	Dexmedetomidine	Vinorelbine	No
DB00535ă	DB00373ă	Cefdinir	Timolol	No
DB01236ă	DB01586	Sevoflurane	Ursodeoxycholic acid	DrugBank
DB01236ă	DB00415ă	Sevoflurane	Ampicillin	No
DB00742ă	DB00441ă	Mannitol	Gemcitabine	DrugBank
DB00585ă	DB01577	Nizatidine	Methamphetamine	DrugBank
DB01136ă	DB00952ă	Carvedilol	Naratriptan	No
DB01203ă	DB01188ă	Nadolol	Ciclopirox	No
DB01204ă	DB00700ă	Mitoxantrone	Eplerenone	DrugBank
DB00968ă	DB00700ă	Methyldopa	Eplerenone	DrugBank
DB01042ă	DB01150ă	Melphalan	Cefprozil	No
DB00519ă	DB00709ă	Trandolapril	Lamivudine	No
DB01026ă	DB00324ă	Ketoconazole	Fluorometholone	No
DB04441ă	DB00224ă	2-Fluoroadenosine	Indinavir	No
DB00441ă	DB01036ă	Gemcitabine	Tolterodine	DrugBank
DB00441ă	DB00438ă	Gemcitabine	Ceftazidime	DrugBank
DB00492ă	DB01115ă	Fosinopril	Nifedipine	DrugBank
DB00472ă	DB00558ă	Fluoxetine	Zanamivir	No
DB00426ă	DB01068ă	Famciclovir	Clonazepam	DrugBank
DB00586ă	DB00790ă	Diclofenac	Perindopril	DrugBank
DB00586ă	DB00745ă	Diclofenac	Modafinil	DrugBank
DB01151ă	DB00501ă	Desipramine	Cimetidine	DrugBank
DB00557ă	DB02300ă	Hydroxyzine	Calcipotriol	No
DB00394ă	DB00459ă	Beclomethasone dipropionate	Acitretin	No
DB00402ă	DB00548ă	Eszopiclone	Azelaic Acid	DrugBank
DB00398ă	DB01223ă	Sorafenib	Aminophylline	DrugBank
DB00346ă	DB00224ă	Alfuzosin	Indinavir	DrugBank
DB01240ă	DB00822ă	Epoprostenol	Disulfiram	No
DB01265	DB00330ă	Telbivudine	Ethambutol	No
DB00659ă	DB00633ă	Acamprosate	Dexmedetomidine	No
DB01033ă	DB00331ă	Mercaptopurine	Metformin	No
DB00503ă	DB00210ă	Ritonavir	Adapalene	No
DB00577ă	DB00953ă	Valaciclovir	Rizatriptan	DrugBank
DB00775ă	DB01006ă	Tirofiban	Letrozole	No
DB00268ă	DB00495ă	Ropinirole	Zidovudine	No
DB01577	DB00585ă	Methamphetamine	Nizatidine	DrugBank
DB00200ă	DB01367ă	Hydroxocobalamin	Rasagiline	DrugBank
DB00555ă	DB01026ă	Lamotrigine	Ketoconazole	DrugBank
DB01195ă	DB00208ă	Flecainide	Ticlopidine	No
DB01019ă	DB00887ă	Bethanechol	Bumetanide	No
DB01053ă	DB00570ă	Benzylpenicillin	Vinblastine	DrugBank
DB00580ă	DB01045ă	Valdecoxib	Rifampicin	DrugBank
DB01265	DB00330ă	Telbivudine	Ethambutol	No
DB00440ă	DB01273	Trimethoprim	Varenicline	DrugBank
DB01162ă	DB00296ă	Terazosin	Ropivacaine	DrugBank
DB01042ă	DB00795ă	Melphalan	Sulfasalazine	DrugBank
DB00648ă	DB00709ă	Lamivudine	Mitotane	DrugBank

Supplementary Table S 7. Investigation of Top 50 predicted DDIs in DrugBank.

The DrugBank evidence list for false positive DDIs is presented in Supplementary Table S7. "No" means that no evidence in DrugBank was found for DDI.

Supplementary File #3 — Two case studies

Case Study 1: Interaction between "DB00798; Gentamicin" and "DB00493; Cefotaxime

Gentamicin has high activity against Gram-positive and Gram-negative bacteria. It is a novel antibiotic compound from Micromonospora[1,2]. It is used for cases with severe infections[3] and treatments of febrile patients that have cancer and granulocytopenia[4].

Cefotaxime is the first choice antibiotics for primary treatment of spontaneous bacterial peritonitis in cirrhosis with serious infections[5,6]. It is verified that Cefotaxime has influence on stimulating growth, regeneration and inducing embryogenesis[7].

A growing body of evidence in the literature confirms the interaction of these two drugs. Some of the literature confirmations are presented here.

- An extensive study on this interaction is done by Bryan *et al*[8], which is published in American Journal of Diseases of Children.
- Murry *et al.*[9] have done a broad investigation of the interaction between these two drugs and proved the resistance to Gentamicin in patients that were consuming Cefatoxime.
- An in vitro studies conducted by Elliott *et al.*[10] suggests that cefotaxime and gentamicin may provide some activity against enterococci.

0.0.1 Case Study 2: Interaction between "DB00945; Acetylsalicylic acid (Aspirin)" and "DB00424; Hyoscyamine"

Acetylsalicylic acid is prescribed in diverse situations for alleviating pain, fever, and inflammation. It can help the patients that had transient ischaemic attack and stroke to decrease the stroke risk[11]. It has some effects on reducing the possibility of the colorectal cancer and some other types of cancer[12].

Hyoscyamine can be found commonly in the stems and leaves of young Datura stramonium plants[13]. It is helpful in the treatment of disorders of the gastrointestinal (GI) tract. It is used in therapies for patients with irritable bowel syndrome, peptic ulcer disease, bladder spasms, colic, pancreatitis, and diverticulitis. Its influence in the treatment of Parkinson is proved[14,15]. Hyoscyamine is effective in short-term tremor abatement[15].

There is an abundant body of evidence for interaction between Aspirin and Hyoscyamine. First, we enquired into "Drugs" database, which is the most popular, wide-ranging and up-to-date source of drug information online[16]. This database declared that Aspirin has an interaction with Hyoscyamine that is available at[17] and also[18]. There is also evidence in "RxList" which is an online medical resource dedicated to offering detailed and current pharmaceutical information on the brand and generic drugs[19]. The information at[20] verified the interaction of mentioned drugs.

Supplementary File #4 — Performance of dimension reduction methods on DS1

We tried to reduce feature dimensions by applying multiple methods and evaluating them. The evaluation criteria in Supplementary Table S8 shows that using common dimension reduction methods does not help the improvement of model.

Method	*Dimension*	*AUC*	*AUPR*	*F − measure*	*Recall*	*Precision*
NMF	600	0.783	0.629	0.617	0.787	0.507
PCA	600	0.802	0.651	0.631	0.729	0.556
SAE	500	0.671	0.504	0.543	0.879	0.393
SAE	128	0.676	0.506	0.545	0.862	0.398
*chi*2	800	0.801	0.655	0.626	0.806	0.512
*chi*2	600	0.786	0.639	0.607	0.885	0.461
*chi*2	300	0.754	0.613	0.577	0.923	0.420

Supplementary Table S 8. Performance of dimension reduction methods on DS1.

Supplementary File #5 — About similarity types

The following information is compiled from the literature and internet.

Similarity measures:

- **Chemical-based**: "Canonical simplified molecular input line entry specification (SMILES) of the drug molecules were downloaded from DrugBank. Hashed fingerprints were computed using the Chemical Development Kit (CDK) with default parameters. The similarity score between two drugs is computed based on their fingerprints according to the two-dimensional Tanimoto score, which is equivalent to the Jaccard score of their fingerprints, that is, the size of the intersection over the union when viewing each fingerprint as specifying a set of elements."[21]

- **Ligand-based**: The Similarity Ensemble Approach (SEA) relates protein receptors based on the chemical 2D similarity of the ligand sets modulating their function. Given a drugs canonical SMILES, the SEA search tool compares it against a compendium of ligand sets and computes E-values for those ligand sets. To compute a drugdrug similarity drugs are queried using their canonical SMILES on the SEA tool. To obtain robust results, the drug is queried against the two ligand databases provided in the tool (MDL Drug data report and WOMBAT) and used two different methods to compute the drug fingerprint (Scitegic ECFP4 and Daylight), resulting in four lists of similar ligand sets. Unifying the four lists and filtering drugligand set pairs with E-values410 _5, a list of relevant protein is obtained receptor families for each drug. Finally, the similarity between a pair of drugs was computed as the Jaccard score between the corresponding sets of receptor families."[21]

- **Side-effect based**: "Drug side effects were obtained from SIDER, an online database containing drug side effects associations extracted from package inserts using text mining methods. This list is augmented by side effect predictions for drugs that are not included in SIDER based on their chemical properties. Following this latter work, the similarity between drugs is defined according to the Jaccard score between either their known side effects or top 13 predicted side effects in case they are unknown."[21]

- **Annotation-based**: The World Health Organization (WHO) ATC classification system is used. "This hierarchical classification system categorizes drugs according to the organ or system on which they act, their therapeutic effect, and their chemical characteristics. ATC codes were obtained from DrugBank. To define a similarity between ATC terms the semantic similarity algorithm of (Resnik, 1999) is used. This algorithm associates probabilities p(x) with all the nodes (i.e., ATC levels) x in the ATC hierarchy by computing the number of levels below x; it then calculates the similarity of two drugs as the maximum over all their common ancestors ATC level c of log (p(c))."[21]

- **Sequence-based**: "Based on a SmithWaterman sequence alignment score between the corresponding drug targets (proteins). Following the normalization suggested in Bleakley and Yamanishi (2009), the SmithWaterman score is divided by the geometric mean of the scores obtained from aligning each sequence against itself."[21]

- **Closeness in a PPI network**: "The distances between each pair of drug targets were calculated using an all-pairs shortest paths algorithm on the human PPI network. Distances were transformed to similarity values using the formula described in Perlman et al (2011):

  $$S(p,p') = Ae^{-D(p,p')} \tag{1}$$

  where $S(p,p')$ is the computed similarity value between two proteins, $D(p,p')$ is the shortest path between these proteins in the PPI network and A was chosen according to Perlman et al (2011) to be 0.9.e. Self-similarity was assigned a value of 1."[21]

- **GO based**: "Semantic similarity scores between drug targets were calculated according to Resnik (1999), using the csbl.go R package selecting the option to use all three ontologies."[21]

- **Off-label side effect**: "OFFSIDES20 is a side effect database built by mining FAERS system while controlling confounding factors such as concomitant medications, patient demographics, and patient medical histories. There are 1,332 drugs and 10,093 side effects in the dataset. We called side effects extracted from OFFSIDES as Off-Label Side Effect[22]. The sets of Off-Label Side Effect of drugs can be vectorized by a binary vector. The similarity of drugs is defined as Jaccard score between either their known Off-Label Side Effects.

- **Target similarity**: "Targets are biological components that drugs interact with and alter their function to induce therapeutic effect(s)"[23]. "If a carrier, transporter, enzyme or target of a given drug is occupied or changed by another drug, then the pharmacological activity of the given drug is changed"[24]. "In order to calculate the similarity between two drugs, we extract the information from drug-target interactions. The underlying assumption made here is that two drugs that share more common target proteins are more similar"[25].

- **Transporter similarity**: "Drug transporters play an important role in modulating drug absorption, distribution, and elimination. Acting alone or in concert with drug metabolizing enzymes they can affect the pharmacokinetics and pharmacodynamics of a drug. This commentary will focus on the potential role that drug transporters may play in drug-drug interactions and what information may be needed during drug development and new drug application (NDA) submissions to address potential drug interactions mediated by transporters"[26]. The Jaccard score between either the known transporters of drug pairs is defined as their similarity.

- **Indication similarity**: "In medical terminology, an "indication" for a drug refers to the use of that drug for treating a particular disease. For example, diabetes is an indication for insulin. Another way of stating this relationship is that insulin is indicated for the treatment of diabet. Medicinenet often have more than one indication, which means that there is more than one disease for which it is used. The Food and Drug Administration (FDA) classifies indications for drugs in the United States"[27]. The Jaccard score between either the known indication of drug pairs is defined as their similarity.

- **Pathways similarity**: 'The pathways of drugs are important information for not only understanding the mechanisms of drug action and metabolism but also for drug repositioning, which finds new therapeutic indications for approved drugs and experimental drugs that fail approval in their initial indication. Therefore, databases collecting drug pathways are increasingly important. Currently, several databases have included drug pathways, such as DrugBank. These databases are useful to drug-related studies. However, most of the drug pathways in the above databases are pathways for drug action and drug metabolism. Actually, besides its targets, one drug can induce expression changes of a number of genes, and thus can deregulate a number of pathways. In recent years, high-throughput technologies such as microarray and RNA-sequencing have produced a lot of drug-induced gene expression profiles. As a result, molecular activity generated from global gene expression profiling now emerges as a promising resource for drug research and then identify the drug-induced pathways"[28]. The Jaccard score between either the known patways of drug pairs is defined as their similarity.

- **Enzymes similarity**: "Enzymes are the proteins in the drug design that act as drug targets for the diseases in the process of drug discovery and development"[29]. The majority of drugs which act on enzymes act as inhibitors and most of these are competitive, in that they compete for binding with the enzyme's substrate- for example the majority of the original (first generation) kinase inhibitors bind to the ATP pocket of the enzyme. Some inhibitors are non-competitive, binding away from the substrate binding domain, competing for co-factor/co-enzyme binding, or causing an allosteric conformational change in the 3-dimensional protein structure that prevents substrate interaction"[30]. The Jaccard score between either the known enzymes that drugs affect them, is defined as their similarity

References

1. Weinstein, M. J. *et al.* Gentamicin, 1 a new antibiotic complex from micromonospora. *J. medicinal chemistry* **6**, 463–464 (1963).
2. Mccracken JR, G., Mize, S., Threlkeld, N. *et al.* Intraventricular gentamicin therapy in gram-negative bacillary meningitis of infancy. *The Lancet* **315**, 787–791 (1980).
3. Prins, J. M., Buller, H. R., Speelman, P., Kuijper, E. & Tange, R. Once versus thrice daily gentamicin in patients with serious infections. *The Lancet* **341**, 335–339 (1993).
4. Schimpff, S., Satterlee, W., Young, V. M. & Serpick, A. Empiric therapy with carbenicillin and gentamicin for febrile patients with cancer and granulocytopenia. *New Engl. J. Medicine* **284**, 1061–1065 (1971).
5. Toledo, C. *et al.* Spontaneous bacterial peritonitis in cirrhosis: predictive factors of infection resolution and survival in patients treated with cefotaxime. *Hepatology* **17**, 251–257 (1993).
6. Felisart, J. *et al.* Cefotaxime is more effective than is ampicillin-tobramycin in cirrhotics with severe infections. *Hepatology* **5**, 457–462 (1985).
7. Mathias, R. J. & Boyd, L. A. Cefotaxime stimulates callus growth, embryogenesis and regeneration in hexaploid bread wheat (triticum aestivum l em. thell). *Plant science* **46**, 217–223 (1986).

8. Bryan, C. S., John, J. F., Pai, M. S. & Austin, T. L. Gentamicin vs cefotaxime for therapy of neonatal sepsis: relationship to drug resistance. *Am. J. Dis. Child.* **139**, 1086–1089 (1985).

9. Murray, P. R. Activity of cefotaxime-aminoglycoside combinations against aminoglycoside-resistant pseudomonas. *Antimicrob. agents chemotherapy* **17**, 474–476 (1980).

10. Elliott, A., Karam, G. H. & Cobbs, C. G. Interaction of cefotaxime and aminoglycosides against enterococci in vitro. *Antimicrob. agents chemotherapy* **24**, 847–850 (1983).

11. Taylor, D. W. *et al.* Low-dose and high-dose acetylsalicylic acid for patients undergoing carotid endarterectomy: a randomised controlled trial. *The Lancet* **353**, 2179–2184 (1999).

12. Patrignani, P. & Patrono, C. Aspirin and cancer. *J. Am. Coll. Cardiol.* **68**, 967–976 (2016).

13. Miraldi, E., Masti, A., Ferri, S. & Comparini, I. B. Distribution of hyoscyamine and scopolamine in datura stramonium. *Fitoterapia* **72**, 644–648 (2001).

14. Sourkes, T. L. " rational hope" in the early treatment of parkinson's disease. *Can. journal physiology pharmacology* **77**, 375–382 (1999).

15. Goetz, C. G. The history of parkinson's disease: early clinical descriptions and neurological therapies. *Cold Spring Harb. perspectives medicine* **1**, a008862 (2011).

16. Drugs. https://www.drugs.com. Accessed 22 Nov 2018.

17. Interaction of aspirin and hyoscyamine, drugs. https://www.drugs.com/drug-interactions/aspirin-low-strength-with-hyoscyamine-methenamine-243-3197-1305-0.html. Accessed 22 Nov 2018.

18. Interactions of hyoscyamine, drugs. https://www.drugs.com/drug-interactions/hyoscyamine-methenamine.html. Accessed 22 Nov 2018.

19. Rxlist. https://www.rxlist.com. Accessed 22 Nov 2018.

20. Interaction of aspirin and hyoscyamine, rxlist. https://www.rxlist.com/drug-interactions/aspirin-diphenhydramine-citrat-oral-and-hyoscyamine-sulfate-oral-interaction.htm. Accessed 22 Nov 2018.

21. Gottlieb, A., Stein, G. Y., Oron, Y., Ruppin, E. & Sharan, R. Indi: a computational framework for inferring drug interactions and their associated recommendations. *Mol. systems biology* **8**, 592 (2012).

22. Zhang, P., Wang, F., Hu, J. & Sorrentino, R. Label propagation prediction of drug-drug interactions based on clinical side effects. *Sci. reports* **5**, 12339 (2015).

23. Campillos, M., Kuhn, M., Gavin, A.-C., Jensen, L. J. & Bork, P. Drug target identification using side-effect similarity. *Science* **321**, 263–266 (2008).

24. Ferdousi, R., Safdari, R. & Omidi, Y. Computational prediction of drug-drug interactions based on drugs functional similarities. *J. biomedical informatics* **70**, 54–64 (2017).

25. Chen, X. *et al.* Nllss: predicting synergistic drug combinations based on semi-supervised learning. *PLoS computational biology* **12**, e1004975 (2016).

26. Zhang, L., Strong, J. M., Qiu, W., Lesko, L. J. & Huang, S.-M. Scientific perspectives on drug transporters and their role in drug interactions. *Mol. pharmaceutics* **3**, 62–69 (2006).

27. medicinenet. https://www.medicinenet.com/indications$for_drugs_{approved_vs_non-approved/views.htm.Lastaccessed}$,2019.

28. Zeng, H., Qiu, C. & Cui, Q. Drug-path: a database for drug-induced pathways. *Database* **2015** (2015).

29. imedpub. http://www.imedpub.com/scholarly/enzymes-and-drug-targets-journals-articles-ppts-list.php. Last accessed, 2019.

30. pharmacologyeducation. https://www.pharmacologyeducation.org/pharmacology/enzymes. Last accessed, 2019.
